# Assessing Ganglion Cell Layer Topography in Human Albinism Using Optical Coherence Tomography

**DOI:** 10.1167/iovs.61.3.36

**Published:** 2020-03-20

**Authors:** Erica N. Woertz, Bisola S. Omoba, Taylor M. Dunn, Stephanie J. Chiu, Sina Farsiu, Sasha Strul, C. Gail Summers, Arlene V. Drack, Joseph Carroll

**Affiliations:** 1 Department of Cell Biology, Neurobiology & Anatomy, Medical College of Wisconsin, Milwaukee, Wisconsin, United States; 2 Medical College of Wisconsin, Milwaukee, Wisconsin, United States; 3 Department of Ophthalmology and Visual Sciences, University of Iowa Hospitals and Clinics, Iowa City, Iowa, United States; 4 Department of Biomedical Engineering, Duke University, Durham, North Carolina, United States; 5 Department of Ophthalmology, Duke University, Durham, North Carolina, United States; 6 Department of Ophthalmology & Visual Neurosciences, University of Minnesota, Minneapolis, Minnesota, United States; 7 Department of Ophthalmology & Visual Sciences, Medical College of Wisconsin, Milwaukee, Wisconsin, United States

**Keywords:** albinism, ganglion cell layer, foveal hypoplasia, optical coherence tomography, foveal development

## Abstract

**Purpose:**

To test whether ganglion cell layer (GCL) and inner plexiform layer (IPL) topography is altered in albinism.

**Methods:**

Optical coherence tomography scans were analyzed in 30 participants with albinism and 25 control participants. Horizontal and vertical line scans were acquired at the fovea, then strip registered and averaged. The Duke Optical Coherence Tomography Retinal Analysis Program was used to automatically segment the combined GCL and IPL and total retinal thickness, followed by program-assisted manual segmentation of the boundary between the GCL and IPL. Layer thickness and area under the curve (AUC) were calculated within 2.5 mm of the fovea. Nasal-temporal and superior-inferior asymmetry were calculated as an AUC ratio in each quadrant.

**Results:**

GCL and IPL topography varied between participants. The summed AUC in all quadrants was similar between groups for both the GCL (*P* = 0.84) and IPL (*P* = 0.08). Both groups showed nasal-temporal asymmetry in the GCL, but only participants with albinism had nasal-temporal asymmetry in the IPL. Nasal-temporal asymmetry was greater in albinism for both the GCL (*P* < 0.0001) and the IPL (*P* = 0.0006). The GCL usually comprised a greater percentage of the combined GCL and IPL in controls than in albinism.

**Conclusions:**

The GCL and IPL have greater structural variability than previously reported. GCL and IPL topography are significantly altered in albinism, which suggests differences in the spatial distribution of retinal ganglion cells. This finding provides insight into foveal development and structure-function relationships in foveal hypoplasia.

The fovea is a region of anatomic specialization in the human retina that is adapted for high-acuity vision. It is characterized by the excavation of inner retinal layers, which forms a pit (reviewed by Provis et al.^1^). The fovea also lacks inner retinal vasculature, forming a region known as the foveal avascular zone. Additionally, foveal cone photoreceptors exhibit increased packing density^2^ and so-called private line circuitry with their midget bipolar cell and midget retinal ganglion cell (mRGC) synaptic partners.^3^^,^^4^ Many hypotheses have been proposed to explain the functional significance of the foveal pit,^5^^–^^7^ but its importance for visual function is best exemplified in human disease. Several pathologic conditions—including albinism,^8^^–^^10^ aniridia,^8^^,^^11^ achromatopsia,^12^^,^^13^ idiopathic congenital nystagmus,^14^ and premature birth^15^^,^^16^—are associated with foveal hypoplasia, in which the foveal pit is underdeveloped or absent, and are also associated with pronounced deficits in visual acuity.

Albinism is a family of genetic diseases that disrupt melanin synthesis and/or cellular trafficking in the retina and often in the skin and hair. Previous studies using optical coherence tomography (OCT) imaging in patients with albinism have demonstrated a wide spectrum of severity of foveal hypoplasia,^10^^,^^17^^–^^19^ which makes it an excellent model to understand the phenotypic range and functional impact of foveal hypoplasia. The most salient feature of foveal hypoplasia is the incomplete excavation of inner retinal layers, including the ganglion cell layer (GCL) and inner plexiform layer (IPL). The GCL is a common target of retinal research because it contains the cell bodies of RGCs, and RGC sampling of visual space is believed to directly limit visual acuity.^20^ The GCL is routinely imaged noninvasively with OCT because this technique is highly accessible in both clinical and research settings, and OCT-based measurements are frequently used clinically to infer RGC density and disease prognosis.^21^^–^^24^ However, owing to low contrast between the GCL and IPL in OCT images, most studies that seek to quantify RGCs using OCT measure the combined GCL and IPL (GCIPL)^21^^,^^23^^,^^25^^,^^26^ or, less commonly, the combined retinal nerve fiber layer (RNFL), GCL, and IPL (ganglion cell complex).^27^ Because the IPL contains RGC dendrites as well as amacrine cells and the RNFL contains RGC axons,^28^ measuring the GCL in combination with neighboring plexiform and/or nerve fiber layers may limit the usefulness of OCT to detect changes in RGC numerosity.

Here, we used OCT to image the fovea and assess inner retinal layer excavation in human albinism, specifically through quantifying the topography of the GCL and IPL. Moreover, we have developed a repeatable method to measure the GCL and IPL independently, rather than the combined GCIPL that is commonly used. We show that these layers have greater structural variability than previously reported and that their topography differs significantly from that observed in control participants. We also discuss the significance of these findings for visual system development in albinism.

## Methods

### Participants

All experiments adhere to the tenets of the Declaration of Helsinki and were approved by the Medical College of Wisconsin Institutional Review Board (PRO 23898). All participants, or participants’ legal guardians if under 18 years old, provided informed consent to the procedures and associated risks and benefits. The participant cohort included 26 control participants with no known history of retinal disease and 55 participants with a clinical diagnosis of albinism. However, 25 participants with albinism were excluded from further analysis because the processed OCT images either did not have sufficient contrast to segment the GCL (*n* = 22) or did not represent the incipient fovea (method for this determination described elsewhere in this article, *n* = 2). Additionally, one control participant and one participant with albinism were excluded because the processed images had a combination of these features. Control participants who were included in the final analysis are described in Table 1, and participants with albinism who were included in the final analysis are described in Table 2. All participants completed an Ocular Health Questionnaire to assess their ocular health history, and axial length was measured using an IOL Master (Carl Zeiss Meditec, Dublin, CA). For participants with albinism, best-corrected visual acuity was measured during a clinic visit or with Early Treatment Diabetic Retinopathy Study charts.

**Table 1.** Summary of Control Participants

				Axial Length (mm)	
Participant	Race	Age (Years)	Sex	OD	OS
JC_0077	White	14	F	24.15	24.11
JC_0200	White	30	M	24.47	24.66
JC_0878	White	12	F	24.03	23.99
JC_0905	White	25	M	22.78	21.98
JC_10312	White	18	M	27.06	26.98
JC_10339	White	29	F	23.54	23.76
JC_10549	White	25	M	24.00	23.89
JC_10567	White	27	F	22.32	22.47
JC_10591	White	26	M	23.56	23.57
JC_11144	Asian	24	M	25.46	25.35
JC_11295	Asian	29	M	22.90	22.94
JC_11314	Black	23	F	24.11	23.84
JC_11321	Asian	29	F	23.71	23.61
JC_11335	White	33	F	24.02	23.86
JC_11341	White	27	F	22.98	23.04
JC_11344	Asian	27	M	24.66	24.78
JC_11350	Black	33	M	23.12	23.20
JC_11354	Native Hawaiian/Pacific Islander	24	F	24.53	24.50
JC_11357	Black	23	M	24.25	24.49
JC_11360	Asian	28	F	23.57	23.58
JC_11364	White	32	F	25.86	25.95
JC_11367	Black	22	M	25.33	25.34
JC_11412	White	23	M	25.32	25.35
JC_11442	White	24	M	23.70	23.63
JC_11617	White	48	F	24.06	23.77

F, female; M, male; OD, right eye; OS, left eye.

**Table 2.** Summary of Participants with Albinism

				Axial Length (mm)		BCVA (logMAR)^*^	
Participant	Race	Age (years)	Sex	OD	OS	OD	OS
JC_0131	Unknown	20	M	24.94	24.87	0.28	0.28
JC_0456	Black	19	M	23.62	22.92	0.34	0.54
JC_0492	White	27	F	23.53	23.48	0.14	0.16
JC_0493	White	20	F	22.33	22.23	0.30^*^	0.00^*^
JC_10093	White	22	M	21.40	22.19	0.66	0.56
JC_10193	White	16	M	24.99	25.30	0.24	0.26
JC_10278	White	14	M	22.82	22.42	0.18	0.46
JC_10508	White	37	F	22.18	ND	0.16	0.28
JC_10725	Unknown	21	F	22.14	22.15	0.60	0.58
JC_10726	Unknown	22	M	23.44	21.96	0.82	0.70
JC_10797	Other	15	M	22.65	22.44	0.46	0.46
BB_10965	White	44	F	23.91	24.99	0.26	0.24
GS_10979	White	17	M	24.10	24.04	0.08	0.26
JC_11046	White	37	M	26.02	25.33	0.54	0.66
GS_11148	White	9	F	21.31	20.88	0.24	0.60
JC_11430	White	16	F	23.40	22.15	0.28	0.32
GS_11807	White	37	M	23.78	24.05	0.70	0.68
JC_11822	White	40	F	21.51	21.55	0.74	0.84
JC_11824	White	12	F	23.22	23.15	0.52	0.54
AD_11837	White	16	M	24.56	24.05	0.16	0.06
JC_11849	White	25	F	22.48	22.34	0.70	0.54
JC_11850	White	33	M	20.15	19.99	0.78	0.88
JC_11851	White	37	F	22.25	21.73	0.72	0.76
JC_11854	Black	33	F	26.97	26.81	0.64	0.56
AD_11897	Black	49	M	21.64	21.71	0.72	0.64
JC_11899	White	13	F	27.37	27.03	0.72	0.58
AD_11925	Unknown	22	M	22.03	22.04	0.58	0.58
JC_11934	White	10	M	23.12	22.91	1.00	1.00
SS_11938	White	22	F	22.40	22.14	0.14	0.22
AD_11941	White	11	M	23.99	24.10	0.32	0.34

BCVA, best-corrected visual acuity; F, female; M, male; ND, not done.

* Visual acuity without correction.

### Image Acquisition

Participants’ pupils were dilated and accommodation was suspended with one drop of phenylephrine hydrochloride (2.5%) followed by one drop of tropicamide (1%). Participants less than 18 years old were given cyclopentolate (1%) in place of tropicamide (1%). OCT images were acquired in all participants using a Bioptigen spectral domain-OCT (Leica Microsystems, Wetzlar, Germany). Volume scans were acquired first to assess the location of the fovea (in controls) or incipient fovea (in participants with albinism). Horizontal and vertical line scans, containing 80 to 100 frames and having nominal lengths of 6 or 7 mm were then acquired at the fovea or incipient fovea. High-quality scans were acquired in both eyes in all control participants and in 15 participants with albinism, and in one eye only for 15 participants with albinism.

### Image Processing

Line scans were loaded into ImageJ^29^ for initial image registration. For each scan (example raw scan shown in Supplementary Video S1), a single reference frame was chosen to register all remaining frames in the scan using the TurboReg plugin,^30^ then the registered scan was saved as a video. That video was then strip-registered as previously described (strip-registered sequence of example scan shown in Supplementary Video S2).^31^ The final processed image was an average of up to 30 frames that had the highest normalized cross-correlation coefficients and met a minimum threshold of 0.85. In some participants, particularly those with pronounced nystagmus, this threshold still permitted inclusion of some frames that were not in perfect register with the chosen foveal reference frame; however, this method allowed inclusion of more frames (or parts of frames) in the average, which provided the necessary signal-to-noise ratio for segmentation of the GCL.

### Foveal Assessment

All processed line scans were assessed by a single reviewer (ENW) to determine whether they accurately represented the fovea or incipient fovea. For control participants, line scans were considered to be foveal if they contained the foveal reflex. For participants with albinism, volume scans were reviewed to determine which frame(s) in the volume scan corresponded with the incipient fovea. This determination was based on features such as inner retinal layer excavation, outer segment elongation, and outer nuclear layer elongation, as previously described.^17^^,^^32^ Next, each processed line scan was compared with the volume scan in order to determine whether the inner retinal topography of the processed image accurately represented the incipient fovea.

For 16 participants included in the final analysis (nine control participants and seven participants with albinism), one of the two line scans from one or both eyes was excluded from analysis because the scans did not accurately represent the fovea or incipient fovea. Additionally, for one participant with albinism (JC_0492), neither line scan from one eye represented the incipient fovea, so only the fellow eye was included in analysis. The scans included in the final analysis for each participant are noted in Supplementary Table S1.

### Segmentation

**Figure 1.**
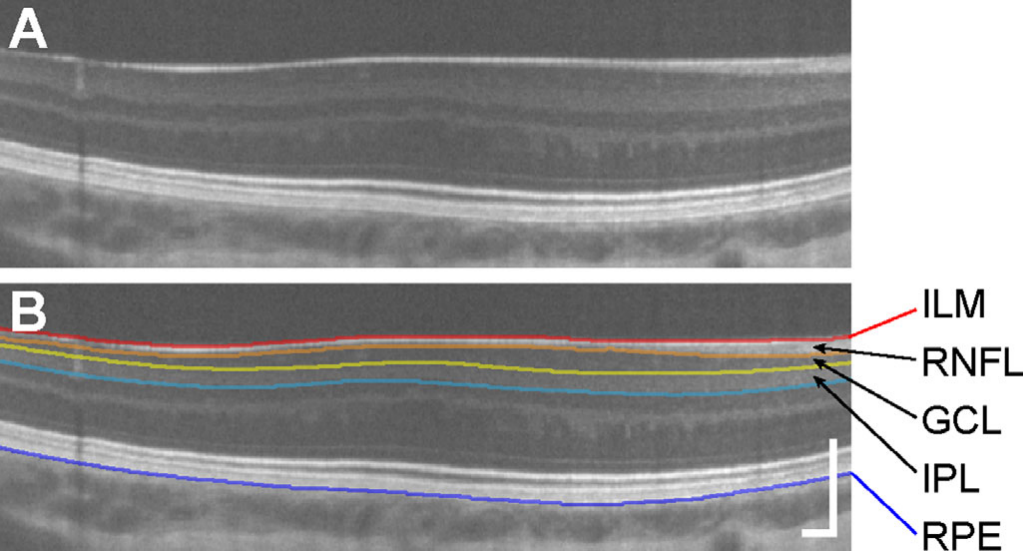
The GCL and IPL can be clearly delimited in processed OCT scans. An example processed image from a participant with albinism, JC_11430 (**A**), is shown with segmentation contours (**B**) for the ILM (*red*), RNFL/GCL boundary (*orange*), GCL/IPL boundary (*yellow*), IPL/INL boundary (*light blue*), and RPE (*dark blue*). Scale bars = 200 µm.

All processed line scans that were included in analysis were segmented twice by a single trained observer (ENW). Averaged images were loaded into the Duke Optical Coherence Tomography Retinal Analysis Program (DOCTRAP),^33^ and automatic segmentation was performed using the program's built-in algorithm for the following retinal layers: the inner limiting membrane (ILM), RNFL, GCL/IPL, inner nuclear layer (INL), outer plexiform layer, and RPE (Fig. 1). Automatic segmentation was followed by manual correction for errors. The GCL was then segmented manually using the DOCTRAP user interface (example segmentation shown in Fig. 1). The segmentation coordinates, in which each layer includes a *y*-coordinate at every pixel along the *x*-axis of the image, were then exported from DOCTRAP for further calculations.

### Thickness and Area Under the Curve (AUC) Calculation

The vertical (axial) scale of the image coordinates (in µm/pixel) was determined using the manufacturer specifications of the original OCT scan. The horizontal (lateral) scale of each image was corrected using each participant’s axial length, as previously described.^10^ Briefly, the length of each scan was calculated by multiplying the nominal scan length by the ratio of the participant’s axial length to the scanner's assumed axial length (24 mm). This scan length was then divided by the number of A-scans per B-scan used for acquisition (1000). Layer thickness was calculated for the total retinal thickness (TRT, distance between the ILM and the RPE), GCL (distance between the RNFL/GCL boundary and the GCL/IPL boundary), IPL (distance between the GCL/IPL boundary and the IPL/INL boundary), and combined GCIPL (distance between the RNFL/GCL boundary and the IPL/INL boundary). In control participants, the fovea was identified by fitting the TRT with a difference of gaussian function as previously described,^34^^,^^35^ then using the fitted function to identify the location of the minimum TRT. In participants with albinism the position of the incipient fovea was identified manually on the unsegmented processed line scans by two trained observers (ENW and JC), and the average of both observers’ responses was used for further analyses. To facilitate comparison of thickness measurements across all participants, raw thickness measurements within 2.5 mm of the fovea or incipient fovea (as identified) were linearly interpolated at 100-µm increments. The AUC for each layer was calculated using trapezoidal numerical integration of the interpolated thickness measurements.

### Statistical Analysis

Repeatability of the interpolated GCL thickness measurements was assessed by comparing the first and second measurements from both eyes (where available) using the method described by Bland and Altman.^36^^–^^38^ Additional statistical analyses were performed using Prism 8 (GraphPad Software, Inc., La Jolla, CA). The sex distribution of each group was compared using a chi-square test. For continuous data the D'Agostino-Pearson test was used to assess normality, where *P* > 0.05 indicated that data were normally distributed. Interocular symmetry was assessed by comparing measurements from each eye (where available; see Supplementary Table S1) using a two-tailed paired *t*-test. For participants with images from both eyes, one eye was randomly selected for subsequent group comparisons. A two-tailed unpaired *t*-test was used to compare normal data and the Mann-Whitney *U* test was used to compare data that were either not normal or showed significantly different variance between groups. Differences between groups were considered to be significant when *P* < 0.05.

## Results

### Participant Demographics

Twenty-five control participants and 30 participants with albinism were included in the final analyses. The control group was 48.0% female and the albinism group was 46.7% female, which was not significantly different between groups (chi-square, *P* > 0.99). The average age (± SD) was 26.2 ± 6.9 years for control participants and 24.3 ± 11.9 years for participants with albinism, and age was also not significantly different between groups (Mann-Whitney *U* test; *U* = 279.5; *P* = 0.11). The average axial length (± SD) was 24.08 ± 1.05 mm for controls and 23.19 ± 1.63 mm for participants with albinism. Axial length was significantly shorter in the albinism group than the control group (Mann-Whitney *U* test; *U* = 217.5; *P* = 0.007), consistent with previous observations.^39^

### Repeatability of GCL Thickness Measurements

**Figure 2.**
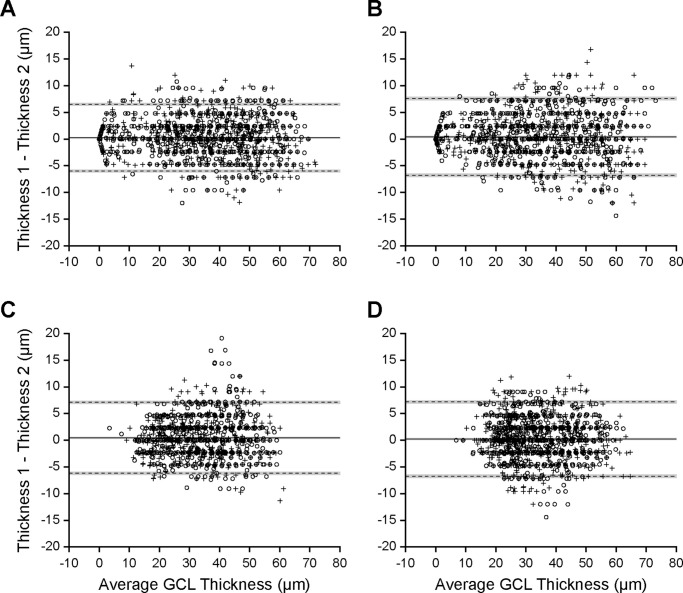
Manual GCL segmentation is repeatable. Bland-Altman plots show repeatability of GCL thickness for control participants in the horizontal (**A**) and vertical (**B**) directions, as well as for participants with albinism in the horizontal (**C**) and vertical (**D**) directions. Each point is a single thickness measurement at a single eccentricity, with 51 measurements per participant per scan. Circles show right eye measurements and crosses show left eye measurements. Solid lines show bias, dashed lines show limits of agreement, and gray boxes show 95% confidence intervals.

The intraobserver repeatability of GCL thickness measurements is shown in Figure 2. Overall, GCL thickness was found to be repeatable for this observer (ENW). For all participants the first measurement was, on average, thicker than the second measurement for both horizontal scans (controls, Fig. 2A: bias ± 95% CI = 0.23 ± 0.13 μm; albinism, Fig. 2C: bias ± 95% CI = 0.47 ± 0.15 μm) and vertical scans (controls, Fig. 2B: bias ± 95% CI = 0.39 ± 0.15 μm; albinism, Fig. 2D; bias ± 95% CI = 0.22 ± 0.15 μm); however, for all scans this bias was less than the height of a single pixel. For all participants, the average of the two measurements was used for further analyses.

### Interocular Symmetry in GCL and IPL Topography

The interocular symmetry of GCL and IPL topography metrics was assessed in participants who had measurements in both eyes using a two-tailed paired *t*-test. The summed four-quadrant AUC was symmetric in all participants for both the GCL (controls: *t* = 0.32, *df* = 15, *P* = 0.75; albinism: *t* = 0.46, *df* = 7, *P* = 0.66) and the IPL (controls: *t* = 0.49, *df* = 15, *P* = 0.63; albinism: *t* = 0.67, *df* = 7, *P* = 0.53). The nasal:temporal AUC ratio, however, was asymmetric for both the GCL (controls: *t* = 3.94, *df* = 22, *P* = 0.0007; albinism: *t* = 2.63, *df* = 11, *P* = 0.02) and the IPL (controls: *t* = 3.25, *df* = 22, *P* = 0.004; albinism: *t* = 2.63, *df* = 11, *P* = 0.02). In controls, nasal-temporal AUC asymmetry was greater in the right eye for the GCL (absolute mean difference = 0.11) and greater in the left eye for the IPL (absolute mean difference = 0.06). In participants with albinism, it was greater in the left eye for the GCL (absolute mean difference = 0.17) and greater in the right eye for the IPL (absolute mean difference = 0.07). In control participants, the superior:inferior AUC ratio was asymmetric for both the GCL (*t* = 7.48, *df* = 17, *P* < 0.0001) and the IPL (*t* = 2.17, *df* = 17, *P* = 0.04), with GCL superior-inferior asymmetry greater in the right eye (absolute mean difference = 0.15) and IPL superior-inferior asymmetry greater in the left eye (absolute mean difference = 0.05). In participants with albinism, the superior:inferior AUC ratio was symmetric for both the GCL (*t* = 0.65, *df* = 9, *P* = 0.53) and the IPL (*t* = 1.48, *df* = 9, *P* = 0.17).

For participants with images from both eyes, one eye was randomly selected for all subsequent comparisons between the control and albinism groups.

### GCL and IPL Thickness Topography

**Figure 3.**
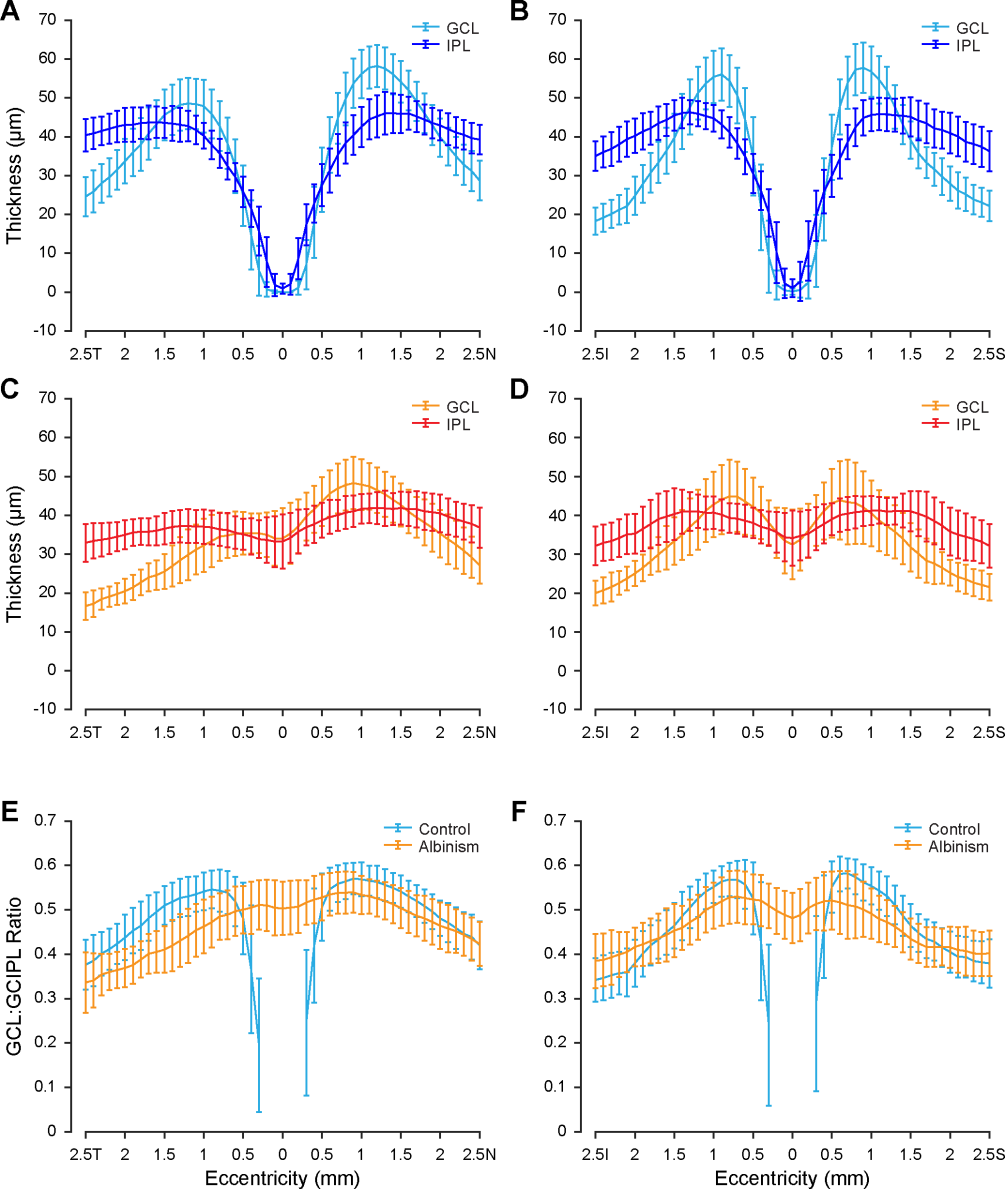
GCL and IPL topography is unique in albinism. GCL and IPL thickness for controls is shown in the horizontal (**A**) and vertical (**B**) directions, as well as for participants with albinism in the horizontal (**C**) and vertical (**D**) directions. The proportional contribution of the GCL to the combined GCIPL is shown for controls and participants with albinism in the horizontal (**E**) and vertical (**F**) directions. Eccentricity directions are labeled temporal (*T*), nasal (*N*), inferior (*I*), or superior (*S*). Error bars show ± 1 SD.

The average topography of GCL and IPL thickness is shown for both controls and participants with albinism in Figure 3. In controls (Figs. 3A, 3B) both GCL and IPL thickness approached zero at the fovea, then rapidly increased with increasing eccentricity until reaching their maximum values near the foveal rim. Both layers then decreased in thickness with increasing eccentricity beyond approximately 1 mm from the fovea. The maximum thickness tended to be further from the fovea in the horizontal direction (Fig. 3A) than in the vertical direction (Fig. 3B).

In participants with albinism (Figs. 3C, 3D) the topography of the GCL and IPL differed qualitatively from that in control participants. Both layers were present at the incipient fovea, which is consistent with foveal hypoplasia. Additionally, within the region examined (i.e., within 2.5 mm from the fovea) the thickness of each layer did not vary as much in the albinism group as in the control group. Notably, in albinism the maximum average GCL thickness in the nasal quadrant was greater than that in the temporal quadrant (Fig. 3C), although it appeared to be more symmetric between the inferior and superior quadrants (Fig. 3D). This nasal-temporal asymmetry was also present among controls (Fig. 3A), but was less pronounced than in albinism.

Differences in layer topography, both between layers (GCL vs. IPL) and between groups (controls vs. albinism), were evident in the percent contribution of the GCL to the GCIPL (Figs. 3E, 3F). In both groups the GCL contribution to the GCIPL was not constant, but rather varied with eccentricity. Within the control group, it was most variable near the fovea but became more consistent with increasing eccentricity. Outside this central-most region (i.e., measurements at least 0.5 mm from the fovea), the GCL contribution to the GCIPL among control participants ranged from 23.4% to 68.5%. Among participants with albinism (across all eccentricities) the GCL contribution ranged from 17.5% to 65.8%. On average, in horizontal scans (Fig. 3E) the GCL comprised a greater percentage of the GCIPL in controls than in participants with albinism (except near the fovea, where the GCL and IPL are not fully excavated in albinism), although there was considerable overlap between groups. In vertical scans (Fig. 3F) the GCL contribution to the GCIPL was only greater in control participants than in participants with albinism near the foveal rim, where the GCL contribution reached its maximum in control participants. However, near the edges of the measured region in vertical scans, the GCL contribution to the GCIPL in albinism approached or exceeded that in controls.

**Figure 4.**
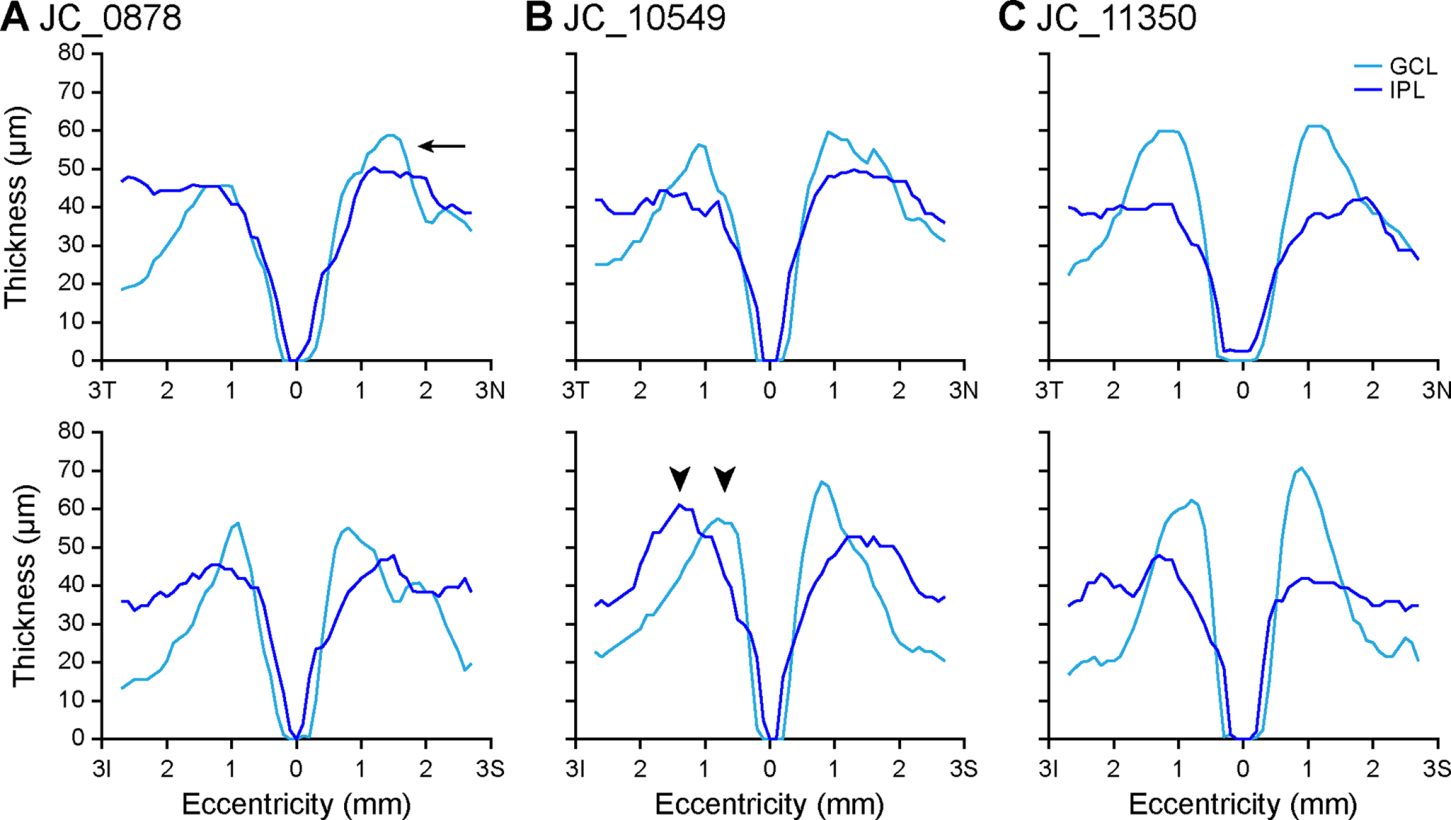
GCL and IPL thickness topography varies among control participants. Some participants, such as JC_0878 (**A**), have GCL thickness that exceeds IPL thickness at the foveal rim in the nasal quadrant (*arrow*), although this may not be the case in the temporal quadrant. This is present in the inferior quadrant in all but seven control eyes, and in the superior quadrant in all but two control eyes. Other participants, such as JC_10549 (**B**), show a pronounced lateral separation between the maximum GCL thickness and maximum IPL thickness, particularly in the inferior quadrant (*arrowheads*). Finally, some participants, such as JC_11350 (**C**), show much greater maximum GCL thickness relative to maximum IPL thickness that is evident in all quadrants. Eccentricity directions are labeled temporal (*T*), nasal (*N*), inferior (*I*), or superior (*S*).

Representative examples of variability in GCL and IPL topography among controls are shown in Figure 4. Although the GCL was usually thicker than the IPL near the foveal rim, in some participants (such as JC_0878; Fig. 4A), this finding was not as pronounced. If this effect was present, it was most likely to be occur in the vertical scan and/or only on the nasal side of the horizontal scan. In other participants (such as JC_10549; Fig. 4B), the maximum GCL thickness occurred much closer to the fovea than the maximum IPL thickness. Finally, in some participants (such as JC_11350; Fig. 4C), the maximum GCL thickness was much greater than the maximum IPL thickness.

### GCL and IPL Area Reflect Cell Layer Topography

**Figure 5.**
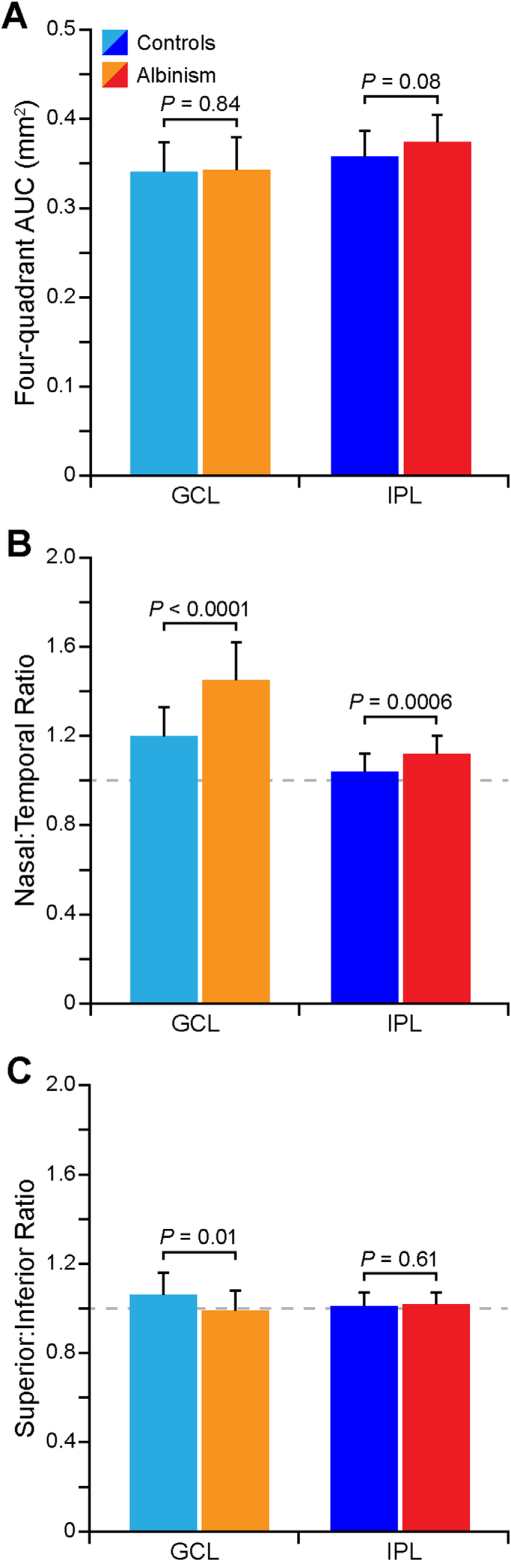
The distribution of GCL and IPL area is unique in albinism. The summed AUC of all four quadrants (temporal, nasal, inferior, and superior) of the GCL and IPL is not different between groups, although the IPL AUC trended toward being greater in albinism (**A**). Both the GCL and IPL show greater nasal-temporal asymmetry in albinism (**B**). The GCL shows greater superior-inferior asymmetry in controls, while the IPL is relatively symmetric in the vertical direction in both groups (**C**).

To assess differences in GCL and IPL topography quantitatively, the AUC for both layers in each quadrant was measured and compared between groups (Fig. 5). The summed GCL AUC for all four quadrants (average ± SD) was 0.341 ± 0.033 mm^2^ for controls and 0.343 ± 0.036 mm^2^ for participants with albinism and was not different between groups (two-tailed *t*-test, *t* = 0.20, *df* = 40, *P* = 0.84). The average summed IPL AUC was 0.358 ± 0.029 mm^2^ for controls and 0.374 ± 0.030 mm^2^ for participants with albinism, which trended toward being greater in albinism than in controls, but this difference was not statistically significant (two-tailed *t*-test, *t* = 1.81, *df* = 40, *P* = 0.08). The ratio between the nasal and temporal quadrants was greater in albinism (GCL = 1.45 ± 0.17, IPL = 1.12 ± 0.08) than in controls (GCL = 1.20 ± 0.13, IPL = 1.04 ± 0.08) for both the GCL (Mann-Whitney *U* test, *U* = 64, *P* < 0.0001) and the IPL (two-tailed *t*-test, *t* = 3.70, *df* = 48, *P* = 0.0006). While this finding shows significantly greater nasal-temporal asymmetry in both layers in albinism, the magnitude of the difference was greater for the GCL than the IPL. The ratio between the superior and inferior GCL AUC was greater in controls (1.06 ± 0.10) than in albinism (0.99 ± 0.09; two-tailed *t*-test, *t* = 2.71, *df* = 45, *P* = 0.01), but was close to unity for both groups, indicating that the GCL was symmetric in vertical scans in both groups The superior:inferior ratio for IPL AUC (controls, 1.01 ± 0.06; albinism, 1.02 ± 0.05) was also symmetric and similar between groups (two-tailed *t*-test, *t* = 0.51, *df* = 45, *P* = 0.61).

### Regular Pattern of Inner Retinal Layer Excavation in Albinism

**Figure 6.**
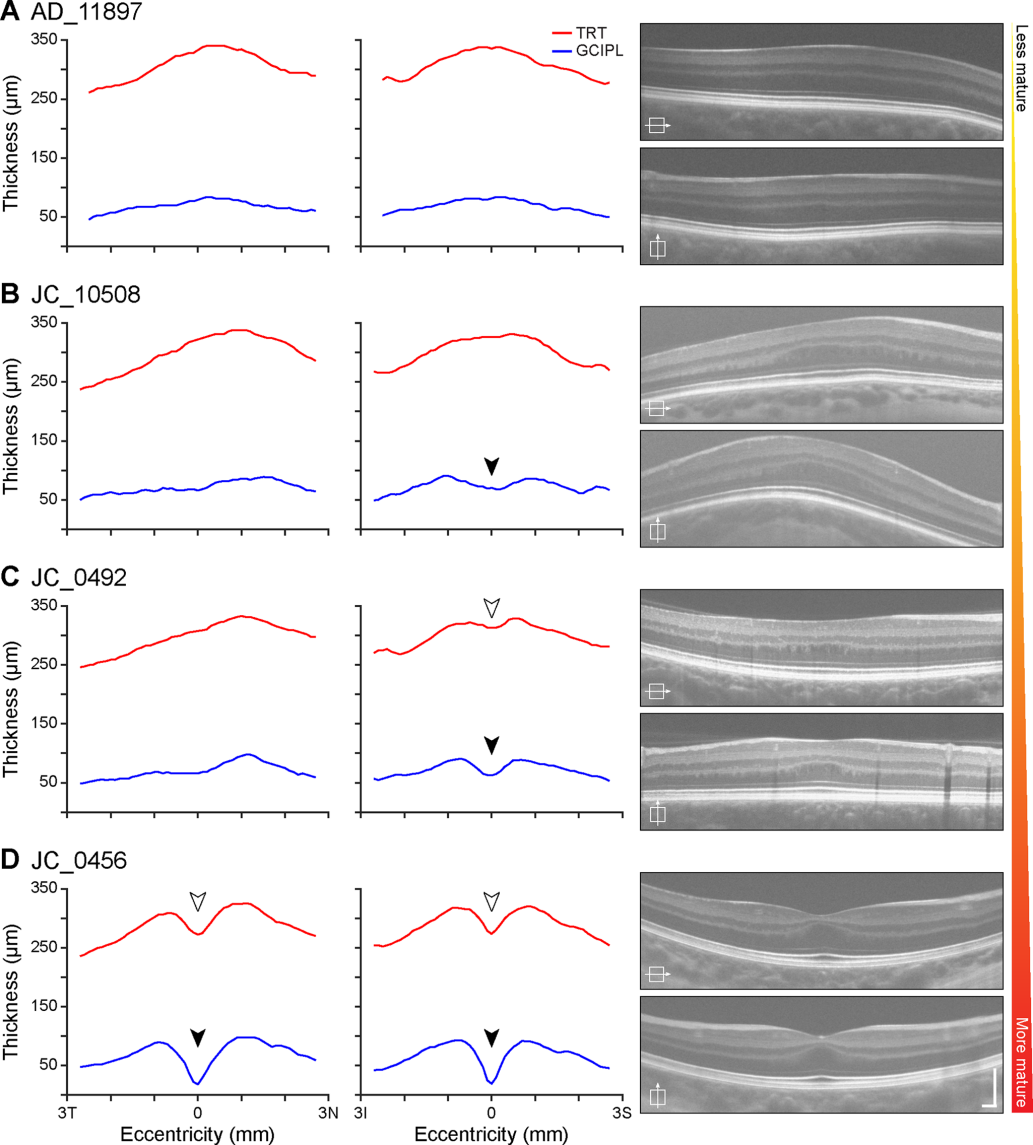
The spectrum of GCL and IPL topography among participants with albinism reflects variability in foveal maturity. Participants with less developed foveae, such as AD_11897 (**A**), show little evidence of a foveal depression. Other participants, such as JC_10508 (**B**) show a depression in the GCIPL (*filled arrowhead*) only in the vertical direction, while participants with slightly more developed foveae, such as JC_0492 (**C**), also show this depression in the TRT (*open arrowhead*). Participants with the most developed foveae, such as JC_0456 (**D**), also show depressions in the GCIPL and TRT in the horizontal direction. Scale bars on OCT images = 200 µm.

Participants with albinism showed a spectrum of foveal development that was reflected in GCL and IPL thickness topography (Fig. 6). Participants who showed the least anatomic specialization had little to no evidence of excavation of the foveal pit, because there was no clear depression either in TRT or in the inner retinal layers (e.g. AD_11897; Fig. 6A). In participants who did show evidence of excavation, this depression was apparent in the GCIPL more frequently than in the TRT, and this commonly occurred only in the vertical scan (compare JC_10508 and JC_0492; Figs. 6B, 6C). Only participants with the most prominent anatomic specialization showed clear excavation of these layers in horizontal scans, and only when it was also present in vertical scans (e.g., JC_0456; Fig. 6D).

## Discussion

Here we show significant differences in the topography of the GCL and IPL in participants with albinism relative to control participants. Foveal hypoplasia is characteristic of albinism and is defined by retention of inner retinal layers (including the GCL and IPL) at the incipient fovea, but this study shows unique features of GCL and IPL topography that have not been previously described. Another study (published during peer review of our study) examined GCL topography in albinism and found a “temporal to central shift” in GCL thickness^40^; thus, our observations largely agree with that study. We expand on the findings of Brücher et al.^40^ by also describing the IPL and examining these layers in a larger participant cohort.

The importance of segmenting the GCL independent of the IPL can be seen in the percent contribution of the GCL to the GCIPL (Figs. 3E, 3F). In both control participants and participants with albinism, the GCL contribution varied widely with eccentricity; thus, the GCL does not comprise a constant proportion of the GCIPL measurement. This finding indicates that the GCIPL is a poor surrogate of GCL thickness topography. Additionally, the wide variability in GCL and IPL topography among control participants (Fig. 4) suggests that there may be additional information to be gleaned about normal foveal structure from parsing these two layers.

### Interocular Asymmetry in GCL and IPL Topography

There was a small but significant difference between eyes for several of the GCL and IPL topography metrics. Because the eye with greater GCL and IPL asymmetry was reversed in albinism relative to controls, it is unlikely that the interocular asymmetry was due to a systematic bias in the image processing or segmentation methods. Additionally, the largest absolute difference between eyes was for the GCL nasal-temporal ratio in albinism (interocular difference of 0.17), but this difference remained less than the average difference in nasal-temporal ratio between albinism and controls (group difference of 0.25). Therefore, we do not believe that interocular asymmetry strongly influences the main finding of greater GCL nasal-temporal asymmetry in albinism relative to controls. This finding is also corroborated by another group who noted a similar effect in albinism even when averaging together measurements from both eyes.^40^

Although interocular asymmetry in the RNFL thickness has been described in normal populations,^41^^,^^42^ it has not been observed in the GCIPL.^41^ The GCL and IPL topography measurements in this study may be more sensitive to interocular asymmetry because they represent area rather than average thickness; thus, slight differences in thickness (which may not be statistically significant on their own) could be multiplied when calculating area. Additionally, the scanning protocol in this study did not account for individual variation in the fovea–Bruch's membrane opening axis angle, which can affect GCIPL intraocular symmetry metrics.^43^ Not only does the fovea–Bruch's membrane opening axis angle vary between individuals but it can also vary between eyes within the same individual,^44^ which could contribute to the interocular asymmetry observed here. In the future, inner retinal layer measurements in albinism may become more precise by measuring and compensating for the fovea–Bruch's membrane opening axis angle.

### GCL and IPL Topography as Indicators of Foveal Development

Inner retinal thickness topography may help to illuminate the sequence of foveal pit formation. Foveal development is believed to be a dynamic process incorporating multiple cellular and physical forces,^45^^–^^47^ and the variation between individuals observed in this study—both among control participants (Fig. 4) and participants with albinism (Fig. 6)—may reflect the complexity of this process. In normal retinal development foveal pit formation is preceded by retinal thickening, or “doming,” at the incipient fovea,^48^ and the nasal-temporal asymmetry in the GCL that was observed in both controls and participants with albinism (Figs. 3A, 3C, and Fig. 5B) could indicate that the location of foveal excavation is slightly temporal to the peak of this dome. The nasal-temporal asymmetry in GCL thickness is also consistent with histological studies in the adult retina that show higher RGC density temporally than in other quadrants.^3^ That this asymmetry was significantly greater in albinism suggests that normal RGC migration is disrupted at some point during foveal development in these patients, leading to even greater asymmetry than is normally observed. Additionally, the differential excavation in horizontal and vertical scans among participants with albinism (Fig. 6) suggests that foveal pit excavation is not symmetric, but rather that cells migrate in different directions at different timepoints throughout the developmental process.

### Relevance for RGC Numerosity and Density in Albinism

Because GCL thickness is frequently used as a surrogate for RGC numerosity,^23^^,^^24^ the area of the GCL and IPL may also provide insight into RGC numerosity and distribution in albinism. Such data are needed to understand the effects of albinism on GCL density, because findings in animal models of albinism vary depending on the species. Studies in Siamese cats and albino ferrets show that RGC numbers are decreased in albinism,^49^^,^^50^ but a study of an albino nonhuman primate found that RGC numbers were normal.^51^ Our study found that GCL area was similar between control participants and participants with albinism, which could indicate that RGC numbers are unchanged in human albinism. Importantly, however, animal models as well as post mortem histology in a human patient have shown that RGC cell bodies are larger in albinism.^49^^,^^51^^–^^53^ If the RGCs are indeed larger, then the number of RGCs per unit area measured on OCT (i.e., RGC density) could be lower than normal; thus, there may still be decreased RGC numbers in albinism (along with altered topographical distribution). To definitively answer this question, further studies in albinism with single-cell resolution of RGCs are needed, which may soon be possible using novel noninvasive, adaptive optics-based imaging methods.^54^^,^^55^

### Impact of Foveal Hypoplasia on Retinal Circuitry and Visual Acuity

Changes in RGC spatial distribution observed in albinism could have implications for RGC circuitry. In the normal retina, foveal cones exhibit private line circuitry with their mRGC partners, in which every cone is connected to both an ON- and an OFF-mRGC; conversely, in the periphery multiple cones converge onto a single mRGC.^3^^,^^4^^,^^56^ This circuitry, taken together with psychophysical studies,^20^ has led to the current view that mRGC sampling is the main determinant of visual acuity thresholds. Additionally, the private line connectivity between cones and their synaptic partners is believed to be necessary to allow foveal cone packing and complete excavation of inner retinal layers.^1^

If the changes in GCL and IPL asymmetry in albinism that are shown in this study do indeed represent altered RGC migration and spatial distribution, then it is feasible that private line circuitry may be disrupted. This finding would be consistent with a recent electron microscopy study in a human retina from an individual who was born prematurely, which showed both foveal hypoplasia and abnormal connectivity between cones and their synaptic partners (Dacey DM, IOVS 2018;59:ARVO E-Abstract 14). This may contribute to impaired inner retinal excavation in foveal hypoplasia and could lead to the decreased visual acuity observed in albinism.^57^^–^^60^ Indeed, foveal hypoplasia grade has thus far proven to be the strongest structural correlate of visual acuity in albinism^60^; thus, foveal structure could be indicative of retinal circuitry. However, the current grading scheme is not a perfect predictor of function, because there is significant overlap in visual acuity ranges between foveal hypoplasia grades.^17^^,^^60^ Moreover, the range of foveal structural specialization observed in albinism overlaps with the normal population.^10^^,^^61^^,^^62^ In the future, continuous metrics of foveal structure may be better equipped to capture the diversity in foveal structure within and between different retinal pathologies and provide greater insight into structure–function relationships in foveal hypoplasia. It is encouraging that some studies have already begun to use continuous metrics to evaluate foveal hypoplasia severity,^14^^,^^63^ and we believe that GCL and IPL topography will provide yet another tool to accomplish this goal.

### Limitations

Owing to the presence of nystagmus in albinism and the necessity for high image quality to complete the analysis, many participants were screened but ultimately excluded from the final analysis. This factor creates the potential for a selection bias. Owing to the wide range of stages of retinal development included in our cohort, we do not think that it had a significant effect on our analysis. If our sample was biased, it is most likely that we lacked adequate representation of more severe foveal hypoplasia, and many of the effects we observed were most prominent in less-developed foveae. Therefore, a less-biased sample would likely increase our effect size rather than decrease it. However, it is possible that there are additional differences between albinism and normal controls that we did not observe in our cohort.

This OCT-based analysis was also restricted to averaged line scans rather than volume scans. Thus, our images only represent cross-sections of the retina along the horizontal and vertical meridians rather than the three-dimensional topography of the entire foveal region. For future studies, volumetric GCL topography would be highly informative, and recently developed techniques for faster and higher contrast OCT imaging—such as visible light OCT^64^^,^^65^ or broadband near-infrared sources^66^—will enable these measurements.

Finally, we found that axial length was significantly shorter in participants with albinism than in controls. It is unclear how differences in axial length may be related to the observed differences in GCL and IPL topography. Because axial length is influenced by many factors—including sex, age, and ethnicity^67^^–^^69^—more work is needed to investigate how changes in axial length are related to other ocular phenotypes in albinism.

## Conclusions

Independent segmentation of the GCL and IPL is critical to understanding inner retinal structure, because it reveals unique topography in each of these layers that is obscured by combined GCIPL measurements. In human albinism, which is characterized by foveal hypoplasia and decreased visual acuity, GCL and IPL thickness topography are significantly altered relative to normal controls. This finding likely indicates differences in the spatial distribution of RGCs, which may affect retinal circuitry. These findings provide greater insight into foveal structure, both in albinism and in normal foveal development, and may also help clarify the structural basis of visual acuity deficits in albinism.

## Supplementary Material

Supplement 1

Supplement 2

Supplement 3

## Supplementary Material

**Supplementary Video S1**. Example raw horizontal OCT scan from a subject with albinism, JC_11849.

**Supplementary Video S2**. Cropped and strip-registered OCT scan from Supplementary Video S1.
