# Supplementary material for: Assessing Ganglion Cell Layer Topography in Human Albinism Using Optical Coherence Tomography
Source: Invest Ophthalmol Vis Sci. 2020 Mar 20;61(3):36. doi: 10.1167/iovs.61.3.36 (PMC7405956; doi:10.1167/iovs.61.3.36)
Supplement: Supplement 1 [file iovs-61-3-36_s001.pdf]

Supplementary Table 1: Line scans included in analysis

Subject	Group	OD* Scans	OS[†] Scans	Selected Eye[‡]
JC_0077	Control	H[§]	H, V[‖]	OS
JC_0200	Control	H, V	H, V	OS
JC_0878	Control	H, V	H, V	OD
JC_0905	Control	H, V	H	OS
JC_10312	Control	H, V	H, V	OS
JC_10339	Control	H, V	H, V	OD
JC_10549	Control	H	H, V	OD
JC_10567	Control	H, V	H, V	OS
JC_10591	Control	H, V	H, V	OS
JC_11144	Control	H, V	H	OS
JC_11295	Control	H, V	H	OD
JC_11314	Control	H, V	H, V	OS
JC_11321	Control	H, V	V	OS
JC_11335	Control	H, V	H	OD
JC_11341	Control	H, V	H, V	OS
JC_11344	Control	H, V	H, V	OD
JC_11350	Control	H, V	H, V	OD
JC_11354	Control	H, V	H, V	OS
JC_11357	Control	H, V	H, V	OS
JC_11360	Control	H, V	V	OS
JC_11364	Control	H, V	H, V	OS
JC_11367	Control	H, V	H, V	OD
JC_11412	Control	H, V	H	OS
JC_11442	Control	H, V	H, V	OS
JC_11617	Control	H, V	H, V	OS
JC_0131	Albinism	H, V	H, V	OS
JC_0456	Albinism	H, V	H, V	OS
JC_0492	Albinism	H, V		OD
JC_0493	Albinism	H, V	H, V	OS
JC_10093	Albinism	H	H, V	OD
JC_10193	Albinism	V		OD
JC_10278	Albinism	H, V		OD
JC_10508	Albinism	H, V		OD

JC_10725	Albinism	H, V	H, V	OD
JC_10726	Albinism	H, V		OD
JC_10797	Albinism	H, V	H, V	OS
BB_10965	Albinism		H, V	OS
GS_10979	Albinism	H, V		OD
JC_11046	Albinism		H, V	OS
GS_11148	Albinism	H, V		OD
JC_11430	Albinism	H, V		OD
GS_11807	Albinism		H, V	OS
JC_11822	Albinism	H, V		OD
JC_11824	Albinism		H, V	OS
AD_11837	Albinism	H	H	OD
JC_11849	Albinism	H, V	H, V	OD
JC_11850	Albinism		H, V	OS
JC_11851	Albinism	H, V	V	OS
JC_11854	Albinism	H, V	H, V	OS
AD_11897	Albinism	H, V	H	OS
JC_11899	Albinism	V		OD
AD_11925	Albinism	H, V	H, V	OS
JC_11934	Albinism		H, V	OS
SS_11938	Albinism	H, V	V	OD
AD_11941	Albinism	H, V	H, V	OD

*OD = right eye; †OS = left eye; ‡Selected Eye = the eye that was used for all monocular analyses; §H = horizontal line scan; ||V = vertical line scan
